# Coalescent Modelling Suggests Recent Secondary-Contact of Cryptic Penguin Species

**DOI:** 10.1371/journal.pone.0144966

**Published:** 2015-12-14

**Authors:** Stefanie Grosser, Christopher P. Burridge, Amanda J. Peucker, Jonathan M. Waters

**Affiliations:** 1 Allan Wilson Centre, Department of Zoology, University of Otago, Dunedin, New Zealand; 2 School of Biological Sciences, University of Tasmania, Hobart, Tasmania, Australia; 3 School of Life and Environmental Sciences, Deakin University, Warrnambool, Victoria, Australia; National Cheng-Kung University, TAIWAN

## Abstract

Molecular genetic analyses present powerful tools for elucidating demographic and biogeographic histories of taxa. Here we present genetic evidence showing a dynamic history for two cryptic lineages within *Eudyptula*, the world's smallest penguin. Specifically, we use a suite of genetic markers to reveal that two congeneric taxa ('Australia' and 'New Zealand') co-occur in southern New Zealand, with only low levels of hybridization. Coalescent modelling suggests that the Australian little penguin only recently expanded into southern New Zealand. Analyses conducted under time-dependent molecular evolutionary rates lend support to the hypothesis of recent anthropogenic turnover, consistent with shifts detected in several other New Zealand coastal vertebrate taxa. This apparent turnover event highlights the dynamic nature of the region’s coastal ecosystem.

## Introduction

Molecular and computational analytical techniques provide important tools for assessing biological history [1, 2], including tests for historic demographic and biogeographic shifts in native species [3, 4]. Such analyses are now key for understanding the histories and trajectories of numerous iconic species e.g., [5, 6]. In addition, the use of these molecular tools has facilitated the discovery of substantial cryptic diversity within many taxa, biodiversity that was previously undetectable using traditional taxonomic approaches [7]. Even within well-known taxa, cryptic species continue to be discovered [8, 9], and researchers have further suggested that many widespread 'species' might actually comprise multiple taxa [10].

Human activity is frequently implicated as a cause of species decline and extinction, but can also facilitate major range shifts in some taxa [5, 11–13]. Such shifts may obscure declines in native species from which the range-expanding species was not previously taxonomically distinguished; the range shift may have contributed to the decline in the native species, or been facilitated by it. For example, the decline of a native Californian blue mussel, *Mytilus trossulus*, was masked by the invasion of the Mediterranean blue mussel, *M*. *galloprovincialis* [11]. Genetic analyses are often key to unravelling such cryptic range shifts.

The little penguin, *Eudyptula minor* (Forster, 1781), endemic to southern Australia and New Zealand (NZ), is the world's smallest extant penguin species [14]. Systematic and taxonomic questions surrounding this taxon have long been controversial. While several subspecies have been recognised based on morphometric and plumage features [15], early molecular analyses failed to support these groupings [16]. In contrast, more recent analyses based on mitochondrial DNA (mtDNA) detected two deeply divergent lineages of little penguin, one restricted to NZ, and another occurring in both Australia and in NZ’s southeast (Otago) [17, 18]. Components of *E*. *minor* vocalisations were also differentiated between Australia and NZ, with Otago birds exhibiting calls similar to those of Australian birds [17]. Based on the deep mtDNA divergence, Banks [17] and Taveres & Baker [19] suggested the possibility of two distinct little penguin species. However, no nuclear DNA evidence has yet been presented to assess the status of these lineages [20], and thus the possibility that *Eudyptula* comprises multiple taxa remains unresolved.

An additional question concerning *E*. *minor* relates to the mechanism whereby these two lineages—potentially distinct species—have come to co-occur in NZ. Recent studies of other vertebrate taxa have revealed several cases of trans-oceanic self-introduction following human impacts [21]. The mtDNA similarity of Otago little penguins to those in Australia was previously attributed to an ancient trans-Tasman dispersal event from Australia to NZ [17, 18] approximately 180,000 years ago (ya) [18]. In contrast, recent discoveries of human-mediated turnover events in southern NZ suggest that anthropogenic forces might instead explain this apparent shift [5, 13]. Under such a scenario, divergence between the Australian and Otago populations would have occurred less than 750 ya [22].

In this study we use a suite of nuclear DNA markers (microsatellites and an intron marker) to test the following hypotheses: (1) that *Eudyptula* comprises two distinct taxa that co-occur in NZ; and (2) that the Australian lineage in Otago represents a recent invasion. We use Markov chain Monte Carlo coalescent analysis to assess timeframes of population divergence.

## Materials and Methods

### Sampling and DNA extraction

**Fig 1:**
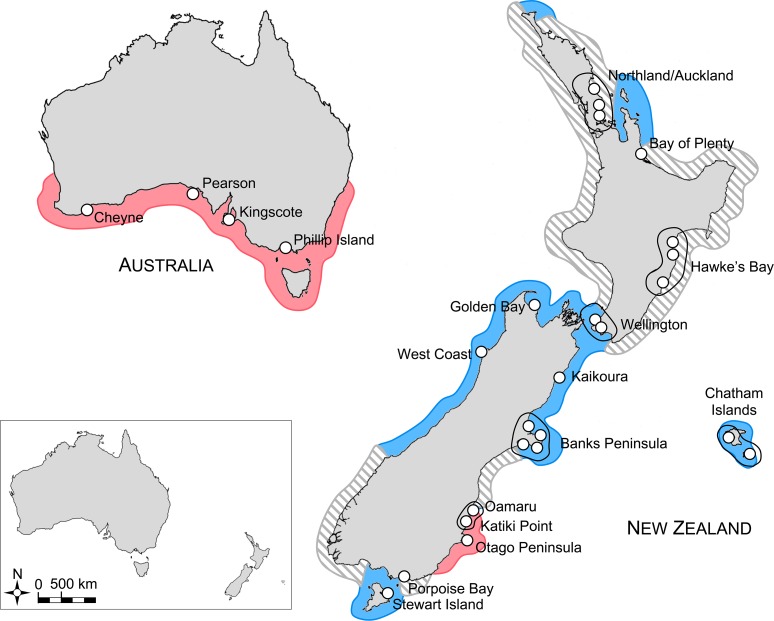
Map of distribution of *Eudyptula* penguins. Blue and red colours indicate previously-inferred ranges of New Zealand and Australian mitochondrial lineages [18,19], respectively. Lineage distributions in grey dashed areas are unknown. White circles mark sampling localities for the current study. Black lines indicate proximate samples pooled as *a priori* regional groupings to increase population sample size.

Frozen or ethanol-preserved samples of *E*. *minor* were collected from localities spanning the distributional range of this species (Fig 1; S1 Table). Samples were collected from beach-wrecked specimens or live sampling at small colonies; individuals sampled or found in the same general geographic area were pooled and considered a population to increase population sample sizes. All samples were collected or held under NZ Department of Conservation (DoC) permit number OT34124-DOA and two variations of this permit. Collection of carcasses did not require an ethics approval. Samples from living birds were received from other research that held independent ethics approvals for their sampling regime. No animals were killed for the purpose of this study. Total DNA was extracted from tissue using a standard 5% Chelex protocol [23]. Alternatively, 5 μL blood samples were extracted using the protocol described in [24]. DNA was also extracted from needles used for insertion of passive integrated transponder (PIT) tags as described in [25]. PIT tags were inserted with permission from DoC under ethics approval no. AEC214 and permit to place transponders into absolutely protected wildlife Authority no. OT – 26993 –FAU. The entire study was approved by the Otago University Animal Ethics Committee.

### Sequencing and genotyping of mitochondrial and nuclear DNA

A 655 bp fragment of the mitochondrial control region HVRI [26] was amplified following [17] with adjusted cycling conditions: initial denaturation at 94°C for 2 min followed by 30 cycles of 94°C for 10 s, 50°C for 10 s, 72°C for 25 s and a final extension at 72°C for 4 min. The PCR product was sequenced using primer ‘D H-Box’ due to the presence of length heteroplasmy (poly-C region) at the opposite end of the fragment (which prevented successful sequencing with CL-tRNAglu) [26]. Sequences were trimmed to a final length of 393 bp. We tested haplotype frequencies for non-neutrality using the Ewens-Watterson-Slatkin test [27] implemented in ARLEQUIN v.3.5 [28] with 10,000 simulations. Ambiguities were coded as missing data in all analyses. Such ambiguities were interpreted as likely reflecting single-site heteroplasmy, as little penguins do not possess a duplicated control region [29]. Analysis of resolved sequences (PHASE v. 2.1 [30]; data not shown) provided no evidence for the existence of nuclear pseudogenes within the data set (e.g., no divergent amplicons from a single sample; no major rate-heterogeneity; broad tree-like structure of sequence data; [31]).

Samples were genotyped at 20 microsatellite loci as described in Grosser & Waters [32]. Microsatellite loci were tested for deviation from Hardy-Weinberg expectations (HWE) and linkage disequilibrium (LD) in each population using GENEPOP v. 4.2.2 [33] with Markov chain parameters of 10,000 dememorisations, 1,000 batches and 10,000 iterations. Corrections for multiple comparisons were carried out to control for False Discovery Rate (FDR) [34] as implemented in the p.adjust function in R. For loci that were inferred to be in LD we attempted to align original 454 sequence reads, from which microsatellite markers where developed (GenBank accessions: KM272221-KM272240), against the chicken and zebra finch genomes (NCBI Genomes database) using NCBI BLAST [35]. Additionally, we used MICRO-CHECKER v. 2.2.3 [36] to test for null-alleles, large allele drop-out and stutter-related miscalling in each population.

The seventh intron of the nuclear β-fibrinogen gene (β-fibint7) was amplified for a spatially representative subset of *E*. *minor* samples (n = 63) using universal primers FIB-BI7U and FIB-BI7L [37]. β-fibint7 was amplified in a 20 μL reaction, containing 10–20 ng of template DNA, 1 × PCR buffer, 1.5 mM MgCl_2_, 200 μM of each dNTP, 0.25 μM of each primer and 0.5 units of *Taq* DNA polymerase (Bioline, London, UK). Cycling parameters consisted of an initial denaturation at 95°C for 5 min, followed by 38 cycles at 95°C for 20 s, 61°C for 25 s and 72°C for 90 s, with a final extension of 72°C for 10 min. Gel purification of PCR products was necessary due to double bands. Bands of approximately 1100 bp were cut from the gel and purified using the Illustra GFX PCR DNA and Gel Band Purification Kit (GE Healthcare, Little Chalfont, UK). PCR fragments were sequenced in both directions, and the alignment was trimmed to 1009 bp. Haplotypes of heterozygous individuals were resolved probabilistically using PHASE v. 2.1 with a phase-probability threshold of 0.95, implemented in DNASP v. 5.10.1 [38]. Sequences were tested for recombination using the Phi test [39] in SPLITSTREE v. 4 [40]. All sequencing and fragment separation was carried out by the Genetics Analysis Service, University of Otago.

### Population structure analysis

We employed a Bayesian clustering approach, implemented in the software STRUCTURE v. 2.3.4 [41] to identify the number of genetic clusters (*K*) of individuals. For the complete dataset containing 477 individuals from all sampling locations and 19 microsatellite loci (with locus Em15 excluded due to null alleles (see Results)) we performed STRUCTURE runs for *K* = 1 to *K* = 10 with 15 iterations for each *K*, a burn-in length of 500,000 followed by 1,000,000 MCMC replications using the admixture and correlated allele frequency models without sampling location information [41, 42]. 95% probability intervals were displayed (ANCESTDIST function turned on) to estimate the error in the calculation of admixture proportions. All other parameters were left at default settings. We estimated the optimal number of clusters following Evanno [43], as implemented in STRUCTURE HARVERSTER Web v. 0.6.93 [44]. CLUMPP v. 1.1.2 [45] was used to average results across the 15 iterations for each STRUCTURE run and final results were visualised using DISTRUCT v 1.1 [46].

### Analysis of genetic variability and population differentiation

Levels of genetic variability for microsatellites and mtDNA were assessed by calculating average number of alleles, expected heterozygosity, observed heterozygosity, numbers of haplotypes, and haplotype and nucleotide diversity in ARLEQUIN. Allelic richness corrected for sample size differences was computed using ADZE v. 1.0 [47].

We quantified levels of genetic differentiation between populations by calculating pairwise Fst and Φst values in ARLEQUIN. Φst values for mtDNA were calculated using Kimura-2-Parameter genetic distances. Significance of Fst and Φst values between populations was tested by permuting haplotypes or alleles between populations (10,000). P-values were adjusted for multiple comparisons. We further tested for hierarchical differentiation within and between the NZ vs. AUS lineages using Analysis of Molecular Variance (AMOVA) [48] performed in ARLEQUIN, based on 10,000 permutations. Median-joining networks were drawn to reconstruct haplotype relationships for mtDNA control region and βcontrol using PopART (http://popart.otago.ac.nz).

Separate analyses on NZ and AUS lineage subsets of the dataset, including Bayesian clustering, AMOVA and Mantel test are described in S1 Appendix.

### Hybrid detection and assignment

We employed two different methods to detect hybrid individuals and evaluate levels of gene flow between *Eudyptula* lineages. First, the assignment method in STRUCTURE was used with 500,000 burn-in, 1,000,000 steps with two groups (AUS and NZ) and migration prior ayText>[47]1. Other parameters were left at default settings. Second, NEWHYBRIDS v.1.1 [49] was run for 500,000 steps after 100,000 burn-in to calculate individual posterior probabilities of being either pure bred to either lineage, a F1 hybrid, a F2 hybrid, or a backcross to either lineage. Results were visualised using DISTRUCT.

### Coalescent analysis

The age of divergence between Otago and Phillip Island populations was assessed under the “Isolation with Migration” framework [50], employing IMa2 [51]. Phillip Island was selected as the genetically closest Australian population for this analysis based on STRUCTURE results (Fig C in S1 Appendix). Mitochondrial, microsatellite, and β-fibint7 markers were analysed concurrently to estimate up to six parameters (two contemporary and one ancestral population size, divergence time of populations, and rates of post-isolation gene flow between populations in each direction). The HKY mutation model was employed for DNA sequence data, and a stepwise mutation model (SMM) for microsatellites (Em2 and Em8 were excluded from the analysis as they exhibited alleles incompatible with SMM). A random subsample of 60 Otago and 20 Phillip Island individuals was selected for analysis (for mtDNA and microsatellites; only eight Otago and four Phillip Island individuals were available for β-fibint7); Otago individuals that represented putative hybrids between AUS and NZ lineages were excluded from this analysis.

Mitochondrial mutation rates were employed in the analysis, against which mutation rates at the nuclear loci would be scaled. Estimates of rates for birds vary widely, perhaps reflecting time-dependency [52, 53]. Here we adopted a conservative approach, using a fast rate (0.86x10^-6^ mutations site^-1^ year^-1^ [54]) derived from Adélie penguin ancient DNA, a pedigree rate (0.55x10^-6^ mutations site^-1^ year^-1^) derived from Adélie penguin family comparisons [54] and a slow rate (0.0295x10^-6^ mutations site^-1^ year^-1^) to explore demographic history. Adélie penguin rates were based on the mtDNA control region. The slower rate estimate was derived from comparisons of cytochrome *b* (cyt *b*) and control region (CR) divergence between the AUS and NZ lineages (4% and 11.8%, respectively [17]). A divergence rate of 2% is often employed for cyt *b* [55]; given the three-fold higher divergence evident in little penguin CR, we calculated a CR-specific divergence rate of 5.9%. For coalescent-derived calibrations we used the associated lineage-specific substitution rate of 2.95%.

We used uniform priors for population size and divergence time parameters, and exponential priors for gene flow, given an expectation that low rates of gene flow were likely. Upper limits on uniform priors of *θ* (= 4*N*_*e*_ *μ*) were set at 20, and the upper limit of the uniform population divergence time prior was set to 1.0. Exponential priors for gene flow were set around a value approximating a mean of 6x10^-6^. Analyses were also carried out under a model assuming no post-divergence gene flow.

An initial run was conducted until stationarity was achieved, assessed based on the lack of trends in posterior parameter estimates, employing MCMC sampling with 80 chains, a geometric chain heating scheme with first and second heating parameters of 0.95 and 0.50, respectively, and eight chain-swap attempts per step. This was then used to seed 24 independent (different random number seed) 24-hour runs each with a short (4 hour) burn-in, but otherwise using the same parameters as above. These 24 runs produced a total of 39,308 (migration) or 38,960 (no migration) genealogies for estimation of model parameters.

## Results

### Identification of genetic clusters

Following FDR correction there was evidence for linkage between some loci, however, the signal was inconsistent between populations and all loci were retained for further analyses. Only one locus (Em15) deviated consistently from HWE after FDR correction and was removed from subsequent analyses (see S1 Appendix for more details).

**Fig 2:**
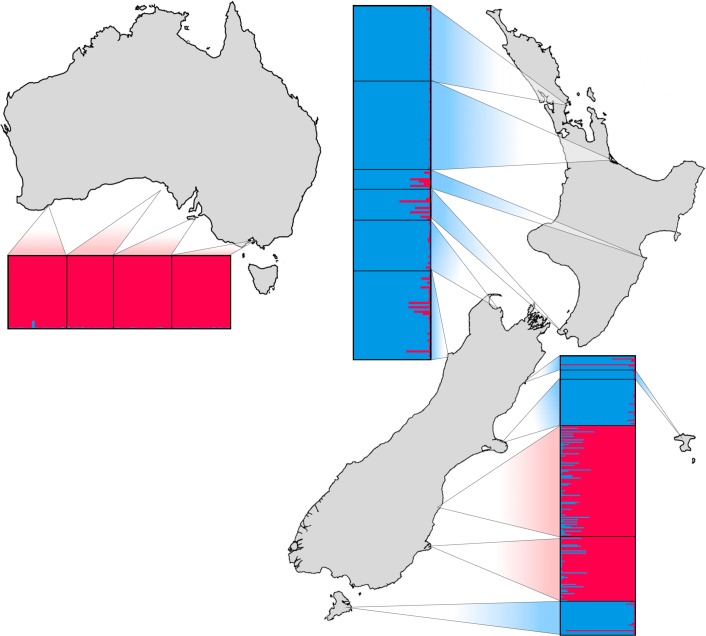
Genetic clustering of *E*. *minor* samples based on STRUCTURE analysis for *K* = 2. Horizontal and vertical bars represent proportional membership of individuals to genetic clusters. Individuals between black lines represent distinct sampling localities as indicated on the map.

Bayesian clustering of individuals suggested two genetically distinct groups, corresponding to the Australian (AUS) and New Zealand (NZ) mtDNA-defined lineages (Fig 2; Evanno and STRUCTURE plots for *K* = 2–5 are shown in S1 and S2 Figs, respectively). Individuals from the four Australian populations showed no admixture with the NZ lineage. Similarly, several NZ populations (Northland/Auckland, Bay of Plenty, Golden Bay, Chatham Islands, and Banks Peninsula) showed no admixture with the AUS lineage (credible intervals for the STRUCTURE plot are displayed in S3 Fig). Two individuals from the NZ locations Kaikoura and Stewart Island showed a high assignment-probability to the AUS lineage. Small numbers of admixed individuals were found in several other NZ locations including Otago, suggesting possible hybridization between these two lineages in NZ (see below).

### Genetic variability and differentiation within and between little penguin colonies and lineages

**Fig 3:**
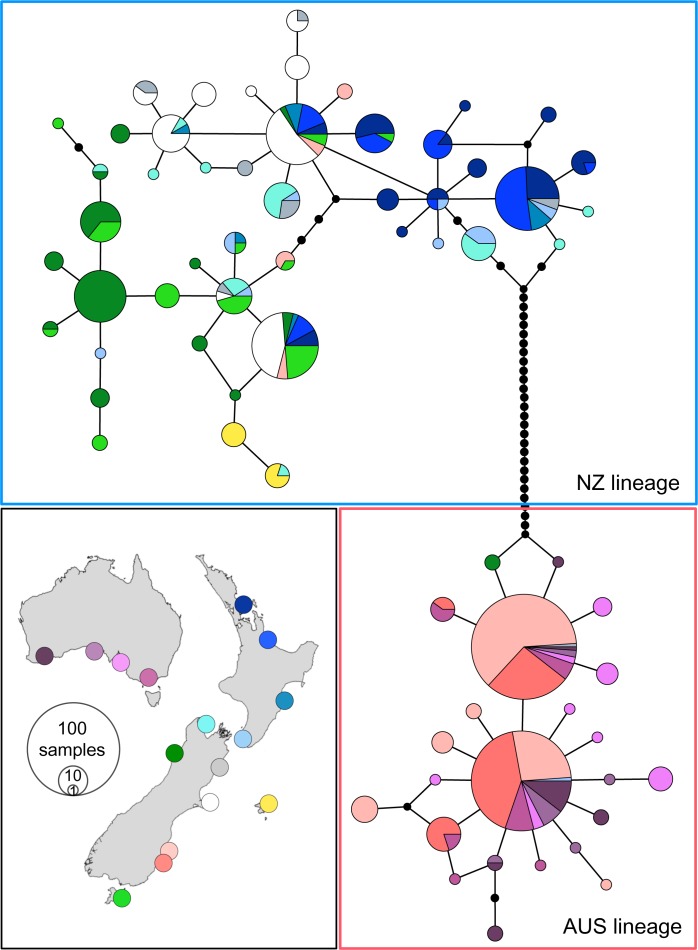
Median-joining network of *Eudyptula* mitochondrial control region haplotypes. Circle size is proportional to haplotype frequency. Colours represent different sampling locations as indicated on the map. Edges between haplotypes represent single mutational steps. Unobserved haplotypes are indicated by small black circles. The red and blue boxes indicate AUS and NZ lineages, respectively.

The Ewens-Watterson-Slatkin test did not reject selective neutrality of mtDNA haplotype frequencies after FDR correction. The Phi test found no evidence for recombination in β-fibint7. The NZ and AUS lineage exhibited similar levels of genetic variation across the 19 microsatellite loci, with mean number of alleles per locus of 9.21 and 9.16, and mean expected heterozygosity of 0.64 and 0.65, respectively. The NZ lineage displayed higher levels of haplotype and nucleotide diversity at the mtDNA control region, 0.98 and 0.024 respectively, compared to 0.86 and 0.013 in the AUS lineage. This difference is also illustrated in the mtDNA haplotype network (Fig 3). Genetic variation of *E*. *minor* lineages and populations is summarised in Table 1.

**Table 1:** Genetic variation within *E*. *minor* samples at 19 microsatellite loci and mtDNA control region.

	Microsatellites							mtDNA			
Location	N	L_poly_	N_a_	A_r_	N_u_	H_e_	H_o_	N	H	h	π
**New Zealand lineage**	**249**	**19**	**9.21**	**8.41**	**33**	**0.64**	**0.62**	**270**	**84**	**0.98**	**0.025**
Northland/Auckland	34	18	6.32	3.44	2	0.61	0.6	34	19	0.95	0.013
Bay of Plenty	40	18	5.95	3.41	3	0.61	0.62	40	13	0.86	0.010
Hawke's Bay	9	18	5	3.65	0	0.65	0.67	10	5	0.8	0.014
Wellington	14	19	5.58	3.55	1	0.64	0.64	14	10	0.96	0.038
Golden Bay	23	18	5.9	3.43	1	0.62	0.63	23	14	0.92	0.020
West Coast	40	18	6.58	3.58	0	0.64	0.63	50	17	0.91	0.020
Kaikoura	12	16^a^	5.74	3.79	1	0.67	0.66	12	7	0.91	0.032
Chatham Islands	8	11^a^	3.74	3.05	1	0.55	0.55	9	3	0.72	0.002
Banks Peninsula	40	18	6.26	3.29	4	0.58	0.58	45	10	0.83	0.005
Stewart Island	29	19	6.11	3.64	2	0.65	0.68	33	15	0.91	0.012
**Australian lineage**	**228**	**19**	**9.16**	**8.39**	**28**	**0.65**	**0.62**	**239**	**44**	**0.86**	**0.014**
Oamaru	96	19	7.37	3.56	3	0.65	0.65	101	13	0.75	0.021
Otago Peninsula	56	18	6.37	3.45	1	0.63	0.64	71	8	0.81	0.005
Phillip Island	20	18	5.53	3.32	3	0.61	0.59	20	7	0.8	0.008
Kingscote	20	19	5.63	3.43	1	0.62	0.62	20	11	0.91	0.009
Pearson	16	11^a^	5.05	3.38	5	0.62	0.57	11	9	0.96	0.009
Cheyne	20	18	5.26	3.33	5	0.62	0.56	16	12	0.96	0.009

N–number of sampled individuals, Lpoly–number of polymorphic loci, Na–mean number of alleles per locus, Ar–allelic richness, Nu–number of private alleles, He–expected heterozygosity, Ho–observed heterozygosity, H–number of haplotypes, h–haplotype diversity, π–nucleotide diversity.

^a^ missing data at locus.

**Fig 4:**
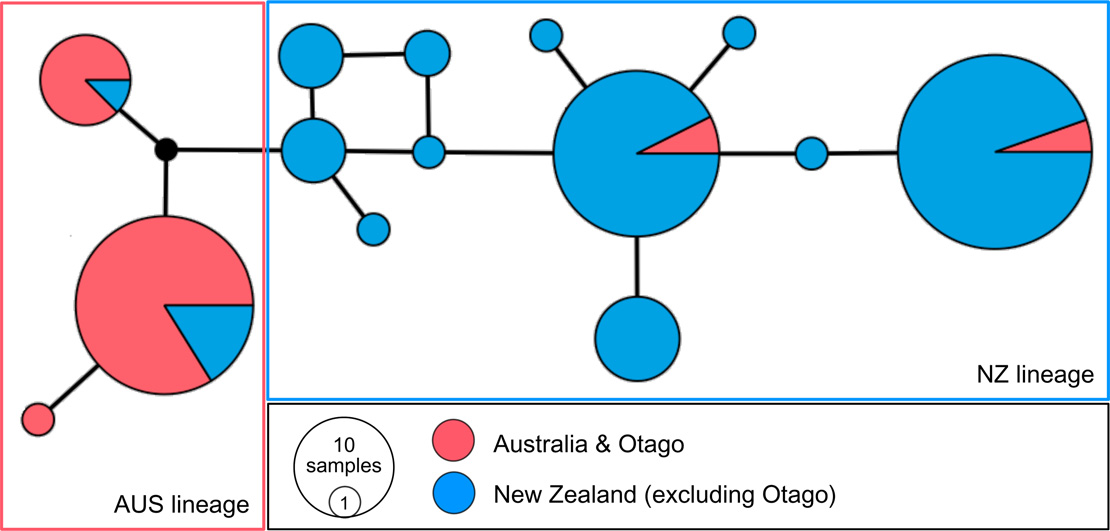
Median-joining network of *Eudyptula* β-fibrinogen intron 7 alleles. Circle size is proportional to allele frequency. Colours represent sampling locations from Australia and Otago (red) and all other NZ locations (blue). Edges between alleles represent single mutational steps. The red and blue boxes contain alleles associated with AUS and NZ lineages of *E*. *minor*, respectively.

The β-fibint7 median-joining network revealed allele groupings and levels of diversity similar to the mtDNA pattern (Fig 4). Several alleles were exclusively or predominantly found in the NZ lineage (Fig 4). While two of these alleles were also detected at low frequencies in the Otago population, the individuals carrying these alleles were identified as hybrids based on mtDNA and microsatellite data. In addition, three alleles were predominantly found in Australian and/or Otago birds. Four individuals from the NZ lineage carrying these ‘Australian’ alleles were also (with only one exception) identified as hybrids.

The NZ and AUS *E*. *minor* lineages exhibited highly significant genetic differentiation, with a Fst value of 0.187 (p < 0.0001) and Φst value of 0.855 (p < 0.0001) for microsatellites and mtDNA control region, respectively. Within the NZ and AUS lineages, many population comparisons yielded significant pairwise Fst and Φst values, and the Chatham Island population was particularly strongly differentiated from all other NZ lineage populations (Table 2). AMOVA indicated high levels of variation among lineages (NZ vs. AUS), with Fct of 0.182 (p < 0.01) for microsatellites and Φct of 0.840 (p < 0.01) for mtDNA (S2 Table).

**Table 2:** Pairwise genetic differentiation among *E*. *minor* samples for microsatellite loci (Fst, above diagonal) and mtDNA control region (Φst, below diagonal).

	New Zealand lineage										Australian lineage					
Location	Nthl	Bop	Hwk	Wel	GB	Wtc	Kai	ChI	BP	SI	Oa	OP	PI	KKS	PS	CW
Nthl		0.002	0.003	**0.031**	**0.026**	**0.027**	**0.018**	**0.110**	**0.043**	**0.031**	**0.208**	**0.227**	**0.252**	**0.262**	**0.256**	**0.258**
Bop	0.009		0.011	**0.045**	**0.035**	**0.034**	**0.034**	**0.108**	**0.055**	**0.040**	**0.209**	**0.231**	**0.254**	**0.261**	**0.254**	**0.254**
Hwk	-0.009	-0.010		-0.001	-0.001	0.004	0.004	**0.113**	**0.030**	0.006	**0.152**	**0.165**	**0.188**	**0.203**	**0.206**	**0.198**
Wel	**0.090**	**0.137**	0.005		**0.015**	**0.018**	0.014	**0.123**	**0.026**	**0.018**	**0.152**	**0.167**	**0.195**	**0.210**	**0.201**	**0.194**
GB	**0.092**	**0.125**	0.009	0.011		**0.010**	-0.004	**0.129**	**0.022**	**0.020**	**0.182**	**0.199**	**0.222**	**0.239**	**0.232**	**0.229**
Wtc	**0.570**	**0.612**	**0.497**	**0.367**	**0.471**		0.008	**0.095**	**0.031**	**0.009**	**0.161**	**0.177**	**0.204**	**0.211**	**0.204**	**0.202**
Kai	**0.074**	**0.082**	-0.027	0.004	0.026	**0.490**		**0.095**	**0.023**	**0.013**	**0.156**	**0.173**	**0.193**	**0.204**	**0.204**	**0.206**
ChI	**0.707**	**0.759**	**0.717**	**0.437**	**0.572**	**0.495**	**0.548**		**0.147**	**0.081**	**0.215**	**0.240**	**0.275**	**0.263**	**0.282**	**0.279**
BP	**0.243**	**0.258**	**0.180**	**0.292**	**0.197**	**0.657**	**0.113**	**0.847**		**0.030**	**0.205**	**0.229**	**0.253**	**0.268**	**0.253**	**0.252**
SI	**0.583**	**0.636**	**0.523**	**0.349**	**0.459**	**0.088**	**0.494**	**0.562**	**0.699**		**0.147**	**0.163**	**0.189**	**0.199**	**0.187**	**0.190**
Oa	**0.859**	**0.864**	**0.847**	**0.805**	**0.844**	**0.850**	**0.813**	**0.862**	**0.881**	**0.867**		**0.010**	**0.017**	**0.033**	**0.024**	**0.033**
OP	**0.946**	**0.950**	**0.954**	**0.914**	**0.937**	**0.920**	**0.926**	**0.966**	**0.964**	**0.949**	**0.047**		**0.023**	**0.048**	**0.051**	**0.052**
PI	**0.923**	**0.932**	**0.927**	**0.844**	**0.899**	**0.887**	**0.867**	**0.956**	**0.957**	**0.928**	0.018	0.012		**0.021**	**0.023**	**0.032**
KKS	**0.921**	**0.931**	**0.923**	**0.842**	**0.896**	**0.885**	**0.864**	**0.949**	**0.956**	**0.925**	**0.187**	**0.370**	**0.296**		0.001	0.008
PS	**0.920**	**0.931**	**0.920**	**0.815**	**0.888**	**0.877**	**0.843**	**0.959**	**0.960**	**0.923**	**0.163**	**0.343**	**0.260**	0.071		0.006
CW	**0.919**	**0.929**	**0.920**	**0.828**	**0.891**	**0.879**	**0.853**	**0.953**	**0.956**	**0.923**	**0.068**	**0.105**	**0.086**	**0.192**	**0.111**	

Values marked in bold were significantly different from zero after correction for multiple comparisons. Nthl–Northland/Auckland, Bop–Bay of Plenty, Hwk–Hawke’s Bay, Wel–Wellington, GB–Golden Bay, Wtc–West Coast, Kai–Kaikoura, ChI–Chatham Islands, BP–Banks Peninsula, SI–Stewart Island, Oa–Oamaru, OP–Otago Peninsula, PI–Phillip Island, KKS–Kangaroo Island, Kingscote, PS–Pearson, CW–Cheyne.

Results from separate analyses on AUS and NZ lineage subsets of the dataset are presented in S1 Appendix.

### Migration and gene flow between little penguin lineages

**Fig 5:**
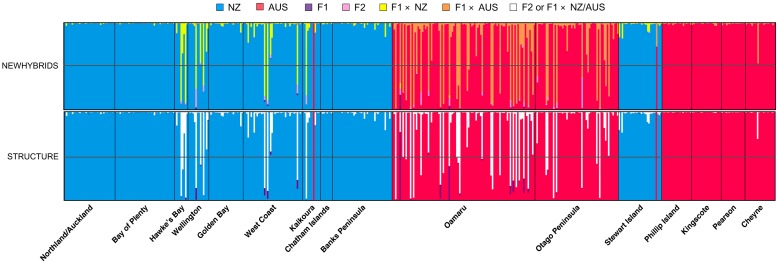
Individual assignment to genetic clusters and hybrid ancestry as determined by STRUCTURE assignment test (bottom plot) and NEWHYBRIDS (top plot). Sampling locations are separated by black vertical lines. Individuals are represented by vertical bars where the colour represents the posterior probability of ancestry of being NZ lineage (blue), AUS lineage (red), a first generation hybrid (F1; purple), a second generation hybrid (F2; pink), a backcross between a F1 and NZ lineage individual (yellow), a backcross between a F1 and AUS lineage individual (orange) or a F2/backcross with either lineage (white; for STRUCTURE plot only). The grey horizontal line indicates 50% posterior probability threshold.

STRUCTURE assignment and NEWHYBRIDS analyses detected two putative AUS-lineage first generation migrants in NZ lineage locations (Kaikoura, Stewart Island), likely sourced from the Otago population (Fig 5). While the Kaikoura individual also carried distinctive AUS mtDNA and β-fibint7 markers, the Stewart Island bird carried a NZ mtDNA haplotype and AUS-lineage β-fibint7, confirming a degree of hybrid ancestry. In addition, STRUCTURE and NEWHYBRIDS analyses detected 10 and nine birds from New Zealand colonies (Hawke’s Bay, Wellington, West Coast, Kaikoura), and 23 and 28 birds from Otago colonies with > 50% posterior probability of mixed ancestry, respectively (Fig 5). With the exception of one potential F1 hybrid, all other individuals with mixed ancestry were most likely backcrosses. Six Otago (Oamaru) birds also carried NZ mtDNA haplotypes. We further found putatively introgressed AUS mtDNA haplotypes and β-fibint7 alleles in NZ birds for which microsatellite-based assignment-tests did not detect hybrid ancestry.

### Coalescent analysis

**Fig 6:**
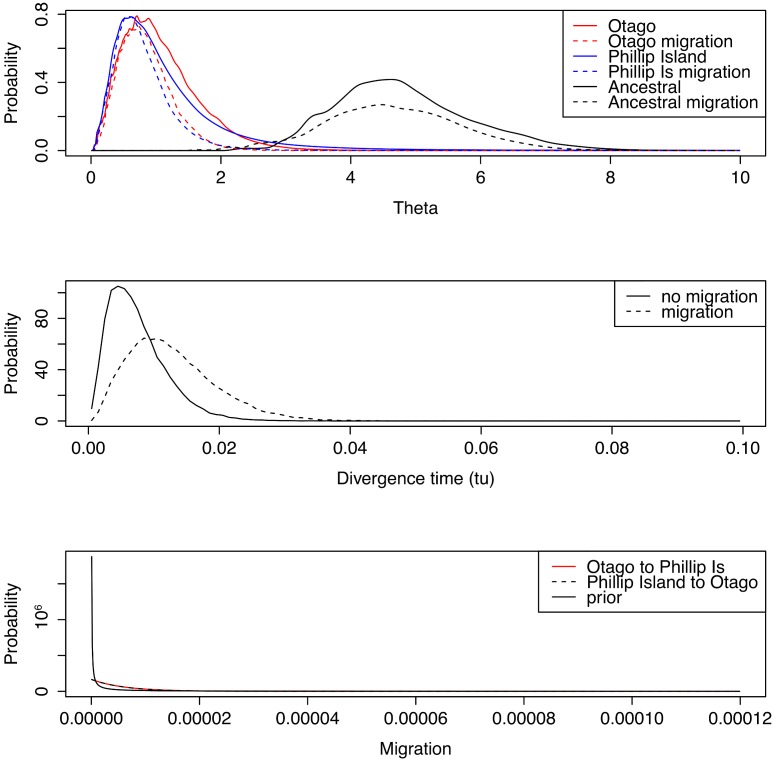
Posterior distributions from analysis under the ‘Isolation with Migration’ model for Otago and Phillip Island populations. Results are presented for analyses with and without post-divergence gene flow (migration). All parameters are scaled by mutation rate. Note that the migration posteriors are described in coalescent terms (movement backwards through time).

Divergence-time estimates for the Otago and Phillip Island populations consistently support a recent (Holocene) isolation event (Table 3), and fail to reject post-human (<750 ya) divergence for the Otago population. Posterior distributions (Fig 6) differed only slightly depending on whether post-divergence migration was included in the model, and there was little evidence for migration when this parameter was included. However, we note that power to detect migration between these recently-diverged lineages may be limited by a lack of diagnostic markers.

**Table 3:** Divergence time estimates t and 95% highest posterior densities (HPD) for Otago and Phillip Island populations based on different mutation rates.

Mutation rate (mutations site^-1^ Myr^-1^)	Source	t in years	95% HPD in years
0.86	ancient DNA [53]	49 (92)	16 (27)–211 (309)
0.55	pedigree data [53]	76 (144)	25 (42)–331 (483)
0.0295	CR/cyt *b* divergence ratio [18]	1422 (2687)	474 (790)–6163 (9008)

Estimates incorporating post-divergence gene flow are shown in parentheses.

## Discussion

Our multilocus genetic analyses of *Eudyptula* penguins confirm that the genus comprises two genetically distinct units. These units broadly correspond to the geographic regions of Australia and NZ, with the exception that the Australian lineage is also present in southern NZ’s Otago region. Multilocus coalescent analyses imply that the Otago population established more recently than previously suggested.

### Differentiation of Eudyptula lineages

Analyses of *Eudyptula* microsatellite markers (19 loci) revealed a clear distinction between NZ and AUS lineages, and thus provide broad genomic support for the previously reported mtDNA-based distinction [17, 18]. Their magnitude of mtDNA control region divergence (10–14%) is clearly comparable to interspecific divergences in *Spheniscus* penguins (8–10%, data not shown). Additionally, Tavares & Baker [19] reported an average sequence divergence between the AUS and NZ lineage at the COI gene of 3.8% compared to only 0.8% between African and Magellanic penguins, and 1.5% between Southern and Northern rockhopper penguins [56], further supporting recognition of multiple *Eudyptula* species.

The β-fibint7 locus revealed less differentiation between the lineages, with some alleles shared between NZ and AUS lineage individuals. While such patterns could reflect either ancestral polymorphism or incomplete lineage sorting [57, 58], the cases of allele sharing between *Eudyptula* lineages typically involved hybrid individuals (as inferred from mtDNA and microsatellite analyses) and thus are most likely attributable to introgression.

In addition to genetic data, there is clear biological evidence supporting a species-level distinction. Feather-colour exhibits significant differentiation between lineages for their blue chroma and maximum brightness [59]. Moreover, vocalisation patterns differ between AUS and NZ lineages [17], and between males from Oamaru (AUS lineage) and Tiritiri Matangi (NZ lineage), with female preference for local calls [60]. Behavioural observations further support a biological distinction between these genetic lineages. Specifically, colonies of the AUS lineage are well-known to come ashore after dusk in 'rafts', i.e., individuals congregate at sea and swim ashore as groups and walk simultaneously to their nesting sites [61, 62]. In NZ, similar rafting behaviour has only been observed in Otago (AUS lineage). This distinctive behaviour may potentially represent a predator avoidance strategy [61]. Interestingly, *Eudyptula* penguins in NZ have not experienced terrestrial vertebrate predators until recently, whereas Australian birds might have regularly encountered carnivorous marsupials [63]. Another distinctive behaviour specific to the AUS lineage (observed in Australia and Otago) is double brooding, i.e., the laying of a second clutch of eggs after a successfully-fledged first clutch [64]. Although double brooding is not found in all Australian colonies, and is thought to be linked to sea-surface temperature and resulting differences in food availability [65, 66], it has never been reported for NZ-lineage colonies despite a similar latitudinal range [67].

### Taxonomic recommendations

Based on our multilocus genetic analyses and concordant biological evidence, we recommend the Australian little penguin to be elevated to full species status, *Eudyptula novaehollandiae* [68], with its type locality restricted to Port Jackson, New South Wales [69]. The NZ little penguin should remain *Eudyptula minor* [70] with the type locality recognised as Dusky Sound, Fiordland. While the divergent mitochondrial and nuclear genetic lineages could alternatively be interpreted as reflecting intraspecific diversity, we feel there is sufficient grounds for them to be considered distinct species. Specifically, these lineages are distinguishable based on phylogenetic, morphological (Grosser, unpublished data) and behavioural features, with evidence for assortative mating, and only limited hybridization despite their occurrence in sympatry for at least a century.

### Extinction-replacement due to anthropogenic causes?

Given controversies around the time-dependency of molecular rates (TDMR) [52, 53, 71], we adopted a conservative approach, using both ‘rapid’ [54], and ‘slow’ rates. Coalescent analyses performed using these rates suggest the Australian and Otago populations diverged substantially more recently (<6200 ya) than previous mtDNA-based estimates (~180 kya) [18]. All three rates indicate recent colonisation timeframes, and all fail to reject a divergence event following human colonisation of NZ (750 ya) [22] when 95% HPD are considered. These findings may parallel human-associated extinction-colonisation events recently inferred for several NZ vertebrate taxa, whereby extirpated mainland lineages were replaced via trans-oceanic colonisation [5, 13].

In addition to coalescent analyses (above), archaeological records provide further evidence of anthropogenic impacts, with substantial *Eudyptula* population declines apparently occurring soon after human settlement. Specifically, while *Eudyptula* is one of the most widely-represented taxa in many of NZ’s pre-human deposits and early archaeological middens, the genus is apparently absent from the majority of late prehistoric midden sites [72]. Based on results from coalescent modelling alone (using different mutation rates and considering the controversy around fast-rate estimates) we cannot completely exclude the possibility that Australian *Eudyptula* colonised Otago before human settlement in NZ. However, given archaeological evidence a post-human arrival for this lineage seems likely. Comprehensive aDNA analyses of fossil and archaeological *Eudyptula* remains similarly support recent (post-human) turn-over of *Eudyptula* lineages in southern NZ (Grosser unpublished data).

### Limited hybridisation between Eudyptula species

Genetic exchange between the Australian and NZ little penguins was restricted to the New Zealand mainland where they co-occur in secondary-contact. It is, however, not yet clear whether introgression of the AUS DNA into NZ populations is exclusively caused by Otago individuals, or if Australian birds occasionally breed in NZ. While the frequency of trans-Tasman dispersal is unknown, it seems reasonable to assume that such events are rare, given that these landmasses are separated by approximately 2,000 km of ocean. Nevertheless, our data demonstrate eastward trans-Tasman migration in the recent past, consistent with prevailing winds and ocean currents e.g., [73, 74]. It is also unclear whether the two apparent first-generation AUS migrants (in Kaikoura and Stewart Island; beach-wrecked specimens) were breeding in these NZ locations or were vagrants. However, it should be noted that birds originally from Oamaru (Otago) have been directly observed breeding in Kaikoura (Agnew, personal communication).

We observed a higher degree of introgression from the NZ species into Otago, matching the theory that a species expanding into the range of a sister taxon will experience greater introgression than the resident species [75]. Nevertheless, the detection of only one F1 hybrid, with all other admixed individuals likely being backcrosses, suggests that levels of gene flow between the species are currently low. This observation might be explained by possible assortative mating, as suggested by female preference of conspecific calls [60] and/or by philopatry [65].

### Conservation implications

Elevation of the Australian little penguin to full species status warrants the reassessment of *Eudyptula* conservation status. While little penguins are globally evaluated as ‘least concern’, their general demographic trend is one of decline [76]. In NZ, most populations are currently considered ‘at risk’ [77], with substantial decline also documented in numerous Australian colonies [78, 79]. Currently, the absence of long-term demographic data precludes an accurate conservation assessment of these species [78].

Our study's findings represent the second recognition of new extant penguin species diversity within a decade [80], and exemplify that cryptic diversity can remain undiscovered even in iconic taxa. Indeed, additional undescribed penguin diversity may exist elsewhere [56]. Our study further highlights the value of genetic approaches for assessing demographic histories of iconic taxa.

## Supporting Information

S1 Appendix Additional information. Separate analyses on NZ and AUS lineage subsets of the dataset.
(DOCX)

S2 Appendix Microsatellite alleles. Provided as an Excel file in GenAlEx format.
(XLS)

S1 Fig STRUCTURE HARVESTER diagrams. Selection of optimal number of clusters *K* for the complete dataset according to the Evanno method.
(TIFF)

S2 Fig Genetic clustering of *Eudyptula minor*. Based on STRUCTURE analysis of the complete dataset for *K* = 2 to *K* = 5. Individuals are represented by vertical bars and colour indicates proportional membership of the individual to a genetic cluster. Black lines separate sampling localities as named below the plot.
(TIFF)

S3 Fig STRUCTURE plot and 95% probability intervals (PI). The middle plot represents the mean values of *q*_*1*_ and *q*_*2*_; the top plot represents the upper PI of *q*_*1*_ and the lower PI of *q*_*2*_; and the bottom plot represents the lower PI of *q*_*1*_ and the upper PI of *q*_*2*_.
(TIFF)

S1 Table Sampling location information. Information on sampling location, sample provider, collection date and tissue type of samples used for population genetic analyses. ^1^samples newly acquired for this study (collected by researcher in connection with other studies); ^2^samples newly acquired for this study; ^3^samples present at the Department of Zoology from previous studies. DoC–New Zealand Department of Conservation.
(DOCX)

S2 Table AMOVA table. Assessment of hierarchical genetic variation between regional *E*. *minor* groupings based on genotypes from 19 microsatellite loci together with sequences from mtDNA control region. NZ–New Zealand, AUS–Australia. Values that were significantly different from zero are indicated by an asterisk.
(DOCX)
