# Supplementary material for: Coalescent Modelling Suggests Recent Secondary-Contact of Cryptic Penguin Species
Source: PLoS One. 2015 Dec 14;10(12):e0144966. doi: 10.1371/journal.pone.0144966 (PMC4682933; doi:10.1371/journal.pone.0144966)
Supplement: S1 Appendix — Separate analyses on NZ and AUS lineage subsets of the dataset. (DOCX) [file pone.0144966.s001.docx]

# S1 Appendix. Additional Information

Coalescent Modelling Suggests Recent Secondary-contact of Cryptic Penguin Species

Stefanie Grosser, Christopher P. Burridge, Amanda J. Peucker and Jonathan M. Waters

# Material and methods

## Population structure analysis

The complete dataset was split into two subsets, one containing Australian and Otago samples (hereafter AUS lineage) and the second containing all other New Zealand samples (NZ lineage), in order to detect more subtle population structures that might have been masked by the stronger signals for the AUS and NZ lineages. For the subsets STRUCTURE was run for *K* = 1 to 10 (NZ) and *K* = 1 to 6 (AUS) with 15 iterations for each *K*, a burn-in length of 500,000 followed by 1,000,000 MCMC replications using the admixture and correlated allele frequency models including sampling location information [[1](#_ENREF_1)].

## Analysis of genetic variability and population differentiation

Population genetic structure within and between defined hierarchical geographic and phylogenetic groups of populations were tested using Analysis of Molecular Variance (AMOVA) performed in ARLEQUIN, based on 10,000 permutations. Specifically, we tested for hierarchical differentiation within and among the following groupings: populations from subspecies ranges as defined by [[2](#_ENREF_2)]; and ad hoc groupings (based on STRUCTURE results and Fst): Chatham Islands vs. all other NZ lineage populations, and northern-most NZ (Northland/Auckland and Bay of Plenty) against all other NZ lineage populations. A Mantel tests was performed in ARLEQUIN based on Fst and shortest coastal distances between NZ lineage sampling locations measured in GOOGLE EARTH v. 7.1.

# Results

## Linkage Disequilibrium and Hardy-Weinberg Equilibrium

Evidence for linkage was found for loci Em9 & Em2, Em8 & Em2, Em5 & Em24 and Em9 & Em13. The signal was inconsistent between populations and in most cases only one population showed significant linkage disequilibrium (LD) after correction for multiple tests. Alignment of sequencing reads to the chicken and zebra finch genomes confirmed possible linkage for Em5 and Em24, both aligning to zebra finch chromosome 4 within approximately 23kb of each other. No match was found for Em9 to confirm possible linkage with Em13. Because there was no consistent pattern of LD across all tested populations we retained all loci for further analysis (we compared results including and excluding Em24 from analyses, due to possible linkage to Em5, but found no difference to the outcome).

Four loci showed significant departure from Hardy-Weinberg equilibrium (HWE) after correction for multiple comparisons. Em15 was consistently deviating from HWE in all New Zealand mainland populations but not in Chatham Island or Australian populations. Em2 showed deviation from HWE at Phillip Island (Australia). Em28 deviated in the Oamaru population and Em6 deviated in Northland/Auckland, Kaikoura and Pearson. Micro-checker suggested null alleles at the deviating loci and also stutter-based miscalling at Em28 and Em6 in Pearson. Because Em15 was consistently deviating from Hardy-Weinberg expectations we removed this locus from further analysis but retained loci that only deviated in few populations.

## Identification of genetic clusters

Separate STRUCTURE analyses for the NZ and AUS lineages using location information as priors yielded small values of r (≤ 0.19) indicating that the incorporation of locality data was highly informative [[1](#_ENREF_1)]. The Evanno method for determining the optimal number of genetic clusters for the NZ lineage dataset was inconclusive (Fig A; STRUCTURE plots for *K*=2-5 are shown in Fig B). The STRUCTURE plot for *K* = 2 reveals a latitudinal cline in genotypic composition, with a gradual north-south genetic transition across central NZ (Fig Ca). In contrast, with *K* = 5, STRUCTURE analysis infers distinct regional groupings associated with northern NZ, central NZ, south-western NZ, Banks Peninsula, and the Chatham Islands (Fig Cb). Within the AUS lineage, STRUCTURE analysis supported two genetically differentiated groupings (Fig Cc), with one cluster containing the three western-most Australian colonies sampled (Cheyne, Pearson and Kingscote), the second containing the Otago (New Zealand) colonies, and Phillip Island representing a mixed assemblage.


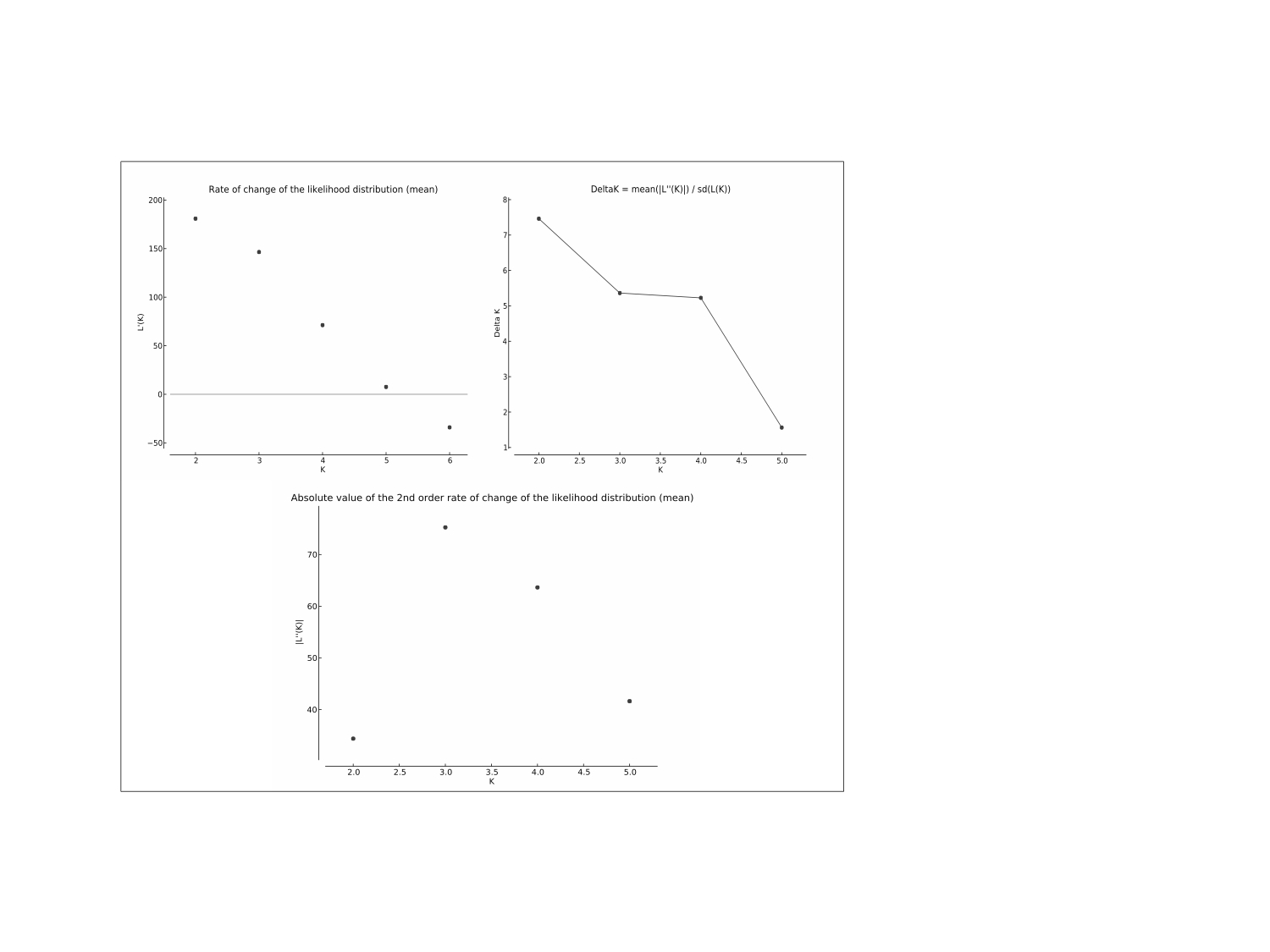


Fig A. STRUCTURE HARVESTER diagrams for selection of optimal number of clusters *K* for the New Zealand populations (excluding Otago) dataset according to the Evanno method [[4](#_ENREF_4)].

Fig B. Genetic clustering of *Eudyptula minor* based on STRUCTURE analysis of the New Zealand populations (excluding Otago) for *K*=2 to *K*=5. Individuals are represented by vertical bars and colour indicates proportional membership of the individual to a genetic cluster. Black lines separate sampling localities as described below the plot.


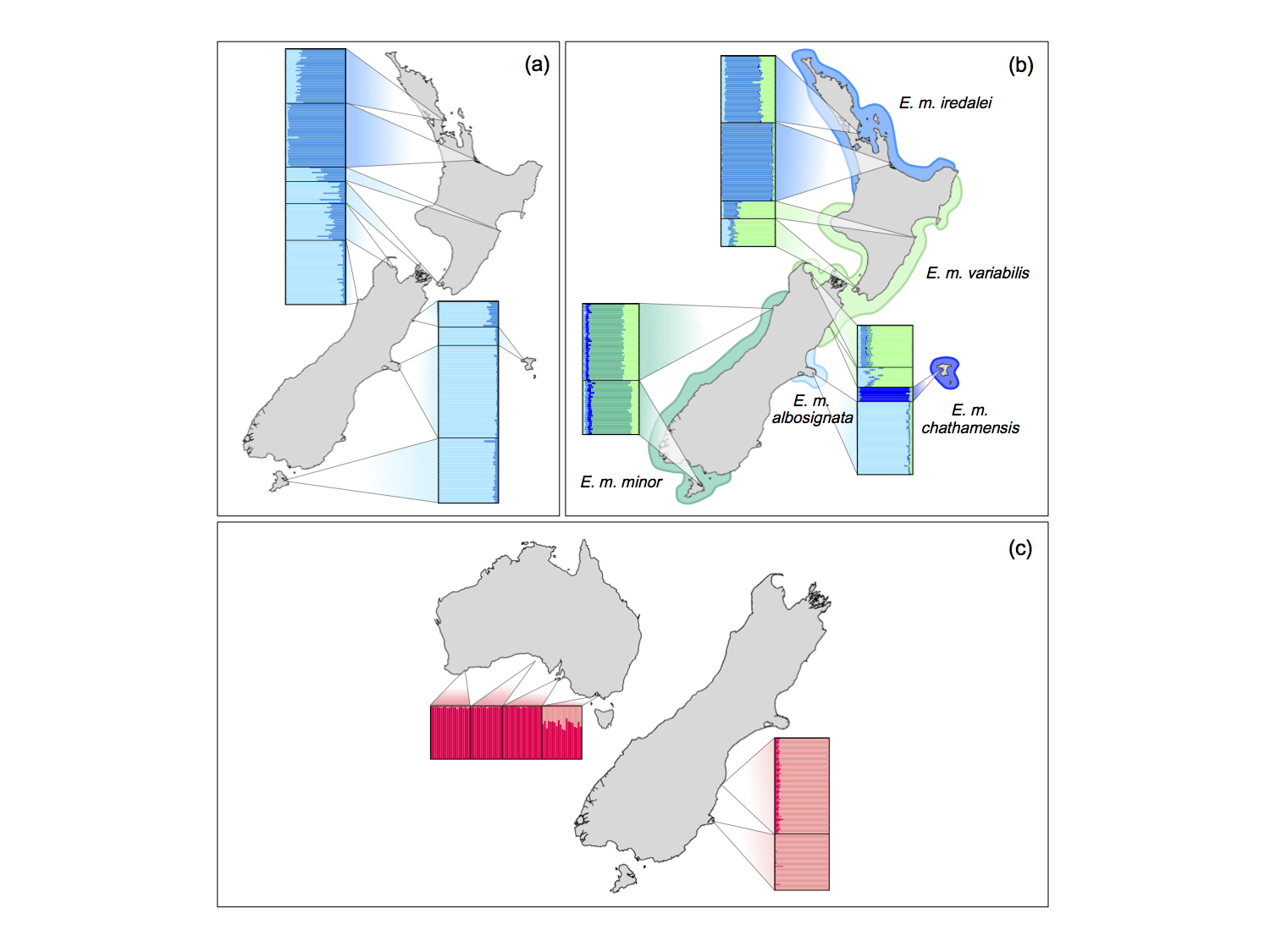


Fig C. Genetic clustering of *E. minor* samples based on STRUCTURE analysis for all New Zealand populations excluding Otago for K = 2 (a); all New Zealand populations excluding Otago for K = 5 (b); and all Australian and Otago populations for K = 2 (c). Horizontal and vertical bars represent proportional membership of individuals to genetic clusters. Individuals between black lines represent distinct sampling localities as indicated on the map. Differently coloured coastal areas (b) represent subspecies ranges described by [[2](#_ENREF_2)].

## Genetic variability and differentiation within and between little penguin colonies and lineages

AMOVA of NZ lineage populations based on subspecies groupings yielded low but significant variation for microsatellite data (Fct = 0.030, p < 0.01), and strong significant variation for mtDNA (Φct = 0.513, p < 0.01; S2 Table). The Mantel test for correlation between geographic distances of NZ lineage colonies and inter-colony Fst was significant for both microsatellites (r = 0.43, p < 0.01) and mtDNA (r = 0.55, p < 0.01); there were too few colonies for a similar test in Australia.

**Discussion**

## Population structure and gene flow within New Zealand E. minor

Significant genetic structuring exists among New Zealand *E. minor* populations. Results of STRUCTURE analysis *K* = 2 indicate largest divergence between northern North Island and the rest of New Zealand, but with some suggestion of a clinal transition. This is supported by significant correlation between geographic and genetic distance, and comparable isolation by distance (IBD) patterns have been previously detected for Australian populations [[3](#_ENREF_3)]. Results for *K* = 5 found groups corresponding to the subspecies ranges defined by [[2](#_ENREF_2)]. AMOVA of subspecies groupings were also significant. The subspecies ranges are, however, distributed in a manner potentially concordant with IBD, and there is thus a possibility that morphological subspecies differentiation simply reflects this IBD. Increased spatial sampling is required to further investigate this issue. Overall the data suggest a low degree of genetic connectivity among some New Zealand populations of little penguins that underlines the importance of conservation management for preserving regional biodiversity.

**References**

1. Hubisz MJ, Falush D, Stephens M, Pritchard JK. Inferring weak population structure with the assistance of sample group information. Mol Ecol Resour. 2009;9: 1322-1332. doi: 10.1111/J.1755-0998.2009.02591.X

2. Kinsky FC, Falla RA. A subspecific revision of the Australasian Blue Penguin (*Eudyptula minor*) in the New Zealand area. Rec Nat Mus New Zeal. 1976;1:105-126.

3. Burridge CP, Peucker AJ, Valautham SK, Styan CA, Dann P. Nonequilibrium conditions explain spatial variability in genetic structuring of little penguin (*Eudyptula minor*). J Hered. 2015. doi: 10.1093/jhered/esv009

4. Evanno G, Regnaut S, Goudet J. Detecting the number of clusters of individuals using the software Structure: a simulation study. Mol Ecol. 2005;14: 2611-2620. doi: 10.1111/J.1365-294x.2005.02553.X
