# Supplementary figures and images for: Coalescent Modelling Suggests Recent Secondary-Contact of Cryptic Penguin Species
Source: PLoS One. 2015 Dec 14;10(12):e0144966. doi: 10.1371/journal.pone.0144966 (PMC4682933; doi:10.1371/journal.pone.0144966)

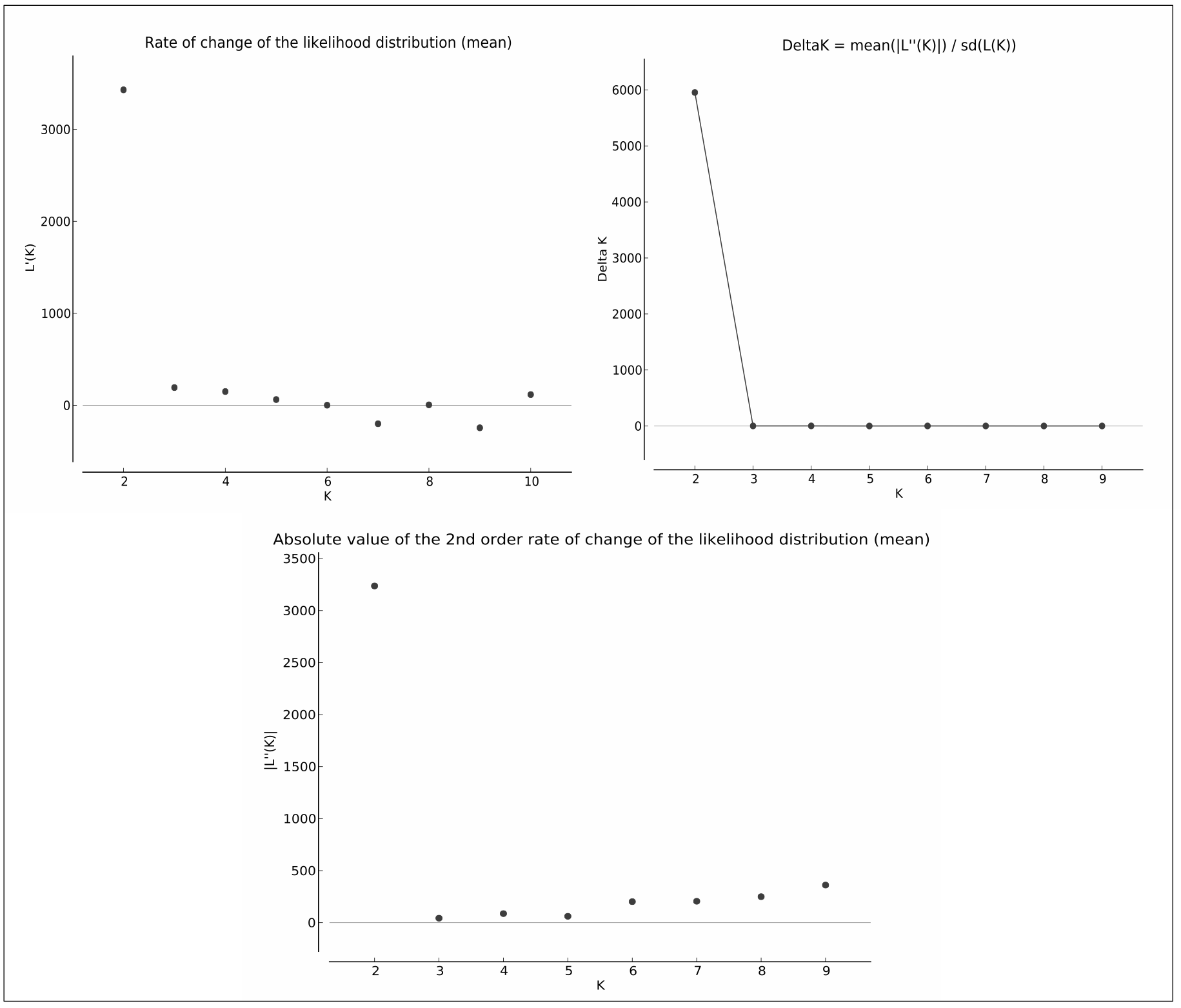

Supplement: S1 Fig — Selection of optimal number of clusters K for the complete dataset according to the Evanno method. (TIFF) [file pone.0144966.s003.tiff]

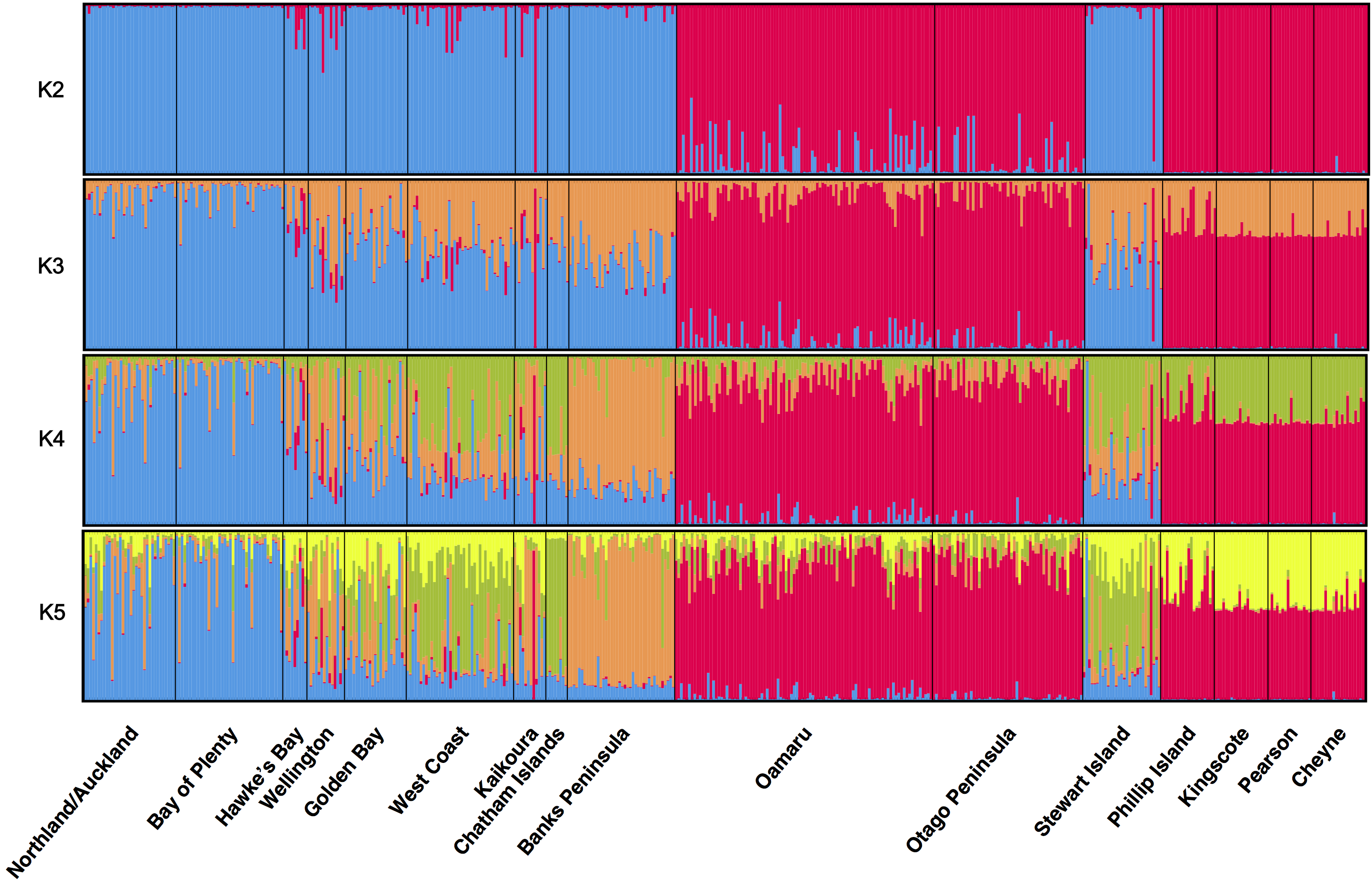

Supplement: S2 Fig — Based on STRUCTURE analysis of the complete dataset for K = 2 to K = 5. Individuals are represented by vertical bars and colour indicates proportional membership of the individual to a genetic cluster. Black lines separate sampling localities as named below the plot. (TIFF) [file pone.0144966.s004.tiff]

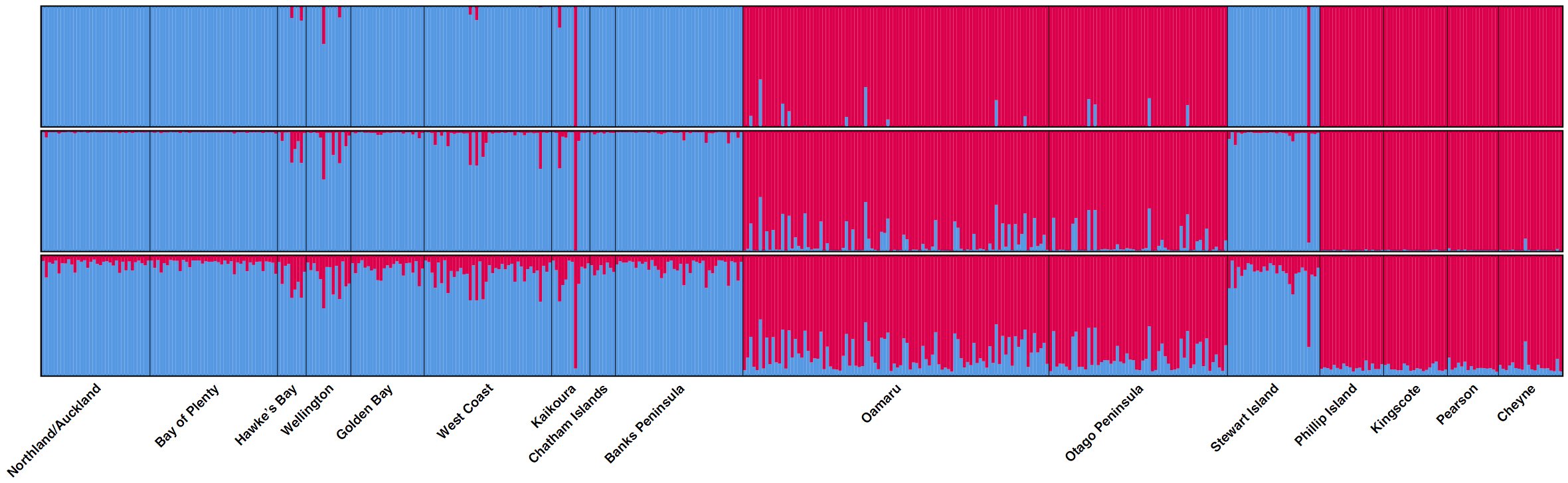

Supplement: S3 Fig — The middle plot represents the mean values of q 1 and q 2; the top plot represents the upper PI of q 1 and the lower PI of q 2; and the bottom plot represents the lower PI of q 1 and the upper PI of q 2. (TIFF) [file pone.0144966.s005.tiff]
