# Supplementary material for: Coalescent Modelling Suggests Recent Secondary-Contact of Cryptic Penguin Species
Source: PLoS One. 2015 Dec 14;10(12):e0144966. doi: 10.1371/journal.pone.0144966 (PMC4682933; doi:10.1371/journal.pone.0144966)
Supplement: S1 Table — Information on sampling location, sample provider, collection date and tissue type of samples used for population genetic analyses. 1samples newly acquired for this study (collected by researcher in connection with other studies); 2samples newly acquired for this study; 3samples present at the Department of Zoology from previous studies. DoC–New Zealand Department of Conservation. (DOCX) [file pone.0144966.s006.docx]

S1 Table. Sampling location information. Information on sampling location, sample provider, collection date and tissue type of samples used for population genetic analyses. ^1^samples newly acquired for this study (collected by researcher in connection with other studies); ^2^samples newly acquired for this study; ^3^samples present at the Department of Zoology from previous studies. DoC – New Zealand Department of Conservation.

| **Region** | **Location** | **Sample provider** | **Provider institution** | **Collection date** | **Sample material** |
| --- | --- | --- | --- | --- | --- |
| Northland/Auckland^1^ | Castor Bay, Goat Island, Hatfield, Leigh, Narrowneck, Northshore, Omaha, Orewa, Roths Bay, , Stanbay, , Takapuna, Tiritiri Matangi, Whangaparoa | Dianne Brunton | Massey University, Auckland | 2010/2011 | Carcasses |
| Bay of Plenty^1(2)^ | Landing Beach, Leisure Island, Mauao, Motiti Island, Mt Maunganui, Mt Motuotau, Piolet Bay, Rabbit Island | Hellen McConnell | Massey University, Palmerston North | 2011/2012 | Blood samples from birds rehabilitated after Rena oil spill |
| Hawke's Bay^1^ | Herbertville, Pandora Pond, Port of Napier, Westshore | Dan Tompkins,  Robyn Hewitt | Landcare Research | 2009 | Genomic DNA (from blood samples) |
| Wellington^2^ | Houghton Bay, Lyall Bay, Makara, Moa Point, Paremata, Plimmerton, Princess Bay, Scorching Bay, Shelley Bay, Somes Is | Lisa Argilla | Wellington Zoo | 2013 | Blood samples from injured and resident birds |
| Golden Bay^3^ | Ligar, Bay, Patons Rock, Pohara, Rangihaeata, Rototai, Tarakohe, Tata Beach, Totaranui, | Mike Ogle | DoC | 2007 | Carcasses (beach wrecked, road kill) |
| West Coast^2^ | Cape Fouldwind, Cape Foulwind quary, Charleston, Fox River, Joyce Bay, Limestone Creek, McCarthy Creek, Nile River, Norfolk Pine, Ranui, Tauranga Bay, White Horse Hill | Reuben Lane | West Coast Blue Penguin Trust | 2009-2012 | Carcasses (beach wrecked, killed by predators) |
| Kaikoura^3^ | Kaikoura car park, Kaikoura coast, Kaikoura Peninsula, Peketa | Mike Morrissey | DoC | 2005-2009 | Carcasses (beach wrecked, road kill) |
| Chatham Islands^3^ | Chatham Island, South East Island | Antje Leseberg | DoC | 2006/2007 | Carcasses |
| Banks Peninsula^3^ | Christchurch Beach (Pegasus Bay), Flea Bay, Harris Bay, Otanerito Beach, Pigeon Bay, Te Oka Bay | Anita Spencer, Chris Challis, Scott Fleming | DoC, independent researcher, University of Otago | 2006/2007/  2010 | Carcasses (beach wrecked, birds killed by predators), freeze-dried blood |
| Oamaru^1^ | Creek colony, Quarry colony | John Cockrem | Massey University, Palmerston North | 2012 | Red blood cells |
| Moeraki^2^ | Harry's Bay, Katiki Point | Rosalie Goldsworthy | Katiki Point Penguin Trust | 2012 | Carcasses (beach wrecked) |
| Otago Peninsula^2^ | Pipikaretu Beach | Hiltrun Ratz | Penguin Place | 2012/2013 | Egg shell membrane (from infertile eggs), PIT tagging needles (and swabs) |
| Porpoise Bay^2^ | Porpoise Bay | Stefanie Grosser | University of Otago | 2013 | Carcass (beach wrecked) |
| Stewart Island^1,3^ | Acker's Point, Half Moon Bay, Horse Shoe Point, Ringa Ringa Beach, | Brent Beavan, Scott Flemming | DoC, University of Otago | 2006/2010 | Carcasses (beach wrecked), freeze-dried blood |
| Phillip Island^1^ | Phillip Island | Amanda Peucker | Deakin University | 2006 | Genomic DNA (from blood samples) |
| Kingscote^1^ | Kingscote | Amanda Peucker | Deakin University | 2006 | Genomic DNA (from blood samples) |
| Pearson^1^ | Pearson | Amanda Peucker | Deakin University | 2006 | Genomic DNA (from blood samples) |
| Cheyne^1^ | Cheyne | Amanda Peucker | Deakin University | 2004 | Genomic DNA (from blood samples) |
