# Supplementary material for: Coalescent Modelling Suggests Recent Secondary-Contact of Cryptic Penguin Species
Source: PLoS One. 2015 Dec 14;10(12):e0144966. doi: 10.1371/journal.pone.0144966 (PMC4682933; doi:10.1371/journal.pone.0144966)
Supplement: S2 Table — Assessment of hierarchical genetic variation between regional E. minor groupings based on genotypes from 19 microsatellite loci together with sequences from mtDNA control region. NZ–New Zealand, AUS–Australia. Values that were significantly different from zero are indicated by an asterisk. (DOCX) [file pone.0144966.s007.docx]

S2 Table. AMOVA table. Assessment of hierarchical genetic variation between regional *E. minor* groupings based on genotypes from 19 microsatellite loci together with sequences from mtDNA control region. NZ – New Zealand, AUS – Australian. Values that were significantly different from zero are indicated by an asterisk.

|  | **Microsatellites**  **(Fst based)** | | |  | **mtDNA**  **(Kimura2P)** | | |
| --- | --- | --- | --- | --- | --- | --- | --- |
| **Source of variation** | Variance | Fixation indices | Per cent variation |  | Variance | Fixation indices | Per cent variation |
|  | **NZ & AUS lineage** | | | | | | |
| Among groups Fct | 1.36 | 0.182* | 18.23 |  | 22.96 | 0.840* | 84.49 |
| Among population within groups Fsc | 0.18 | 0.029* | 2.36 |  | 1.48 | 0.350* | 5.43 |
| Within populations Fst | 5.92 | 0.206* | 79.41 |  | 2.74 | 0.900* | 10.07 |
|  | **NZ lineage subspecies** | | | | | | |
| Among groups Fct | 0.18 | 0.030* | 2.99 |  | 2.53 | 0.513* | 51.33 |
| Among population within groups Fsc | 0.04 | 0.007* | 0.64 |  | 0.19 | 0.080* | 3.92 |
| Within populations Fst | 5.83 | 0.036* | 96.36 |  | 2.17 | 0.552* | 44.76 |
|  | **NZ lineage & Chatham Islands** | | | | | | |
| Among groups Fct | 0.54 | 0.084 | 8.30 |  | 2.12 | 0.326 | 32.58 |
| Among population within groups Fsc | 0.16 | 0.026* | 2.39 |  | 2.22 | 0.507* | 34.15 |
| Within populations Fst | 5.83 | 0.107* | 89.3 |  | 2.17 | 0.667* | 33.27 |
|  | **NZ lineage & Northland/Auckland - Bay of Plenty** | | | | | | |
| Among groups Fct | 0.11 | 0.018* | 1.80 |  | 0.46 | 0.096* | 9.58 |
| Among population within groups Fsc | 0.14 | 0.024* | 2.34 |  | 2.17 | 0.501* | 45.62 |
| Within populations Fst | 5.83 | 0.041* | 95.86 |  | 2.17 | 0.548* | 45.16 |
